# Clinical and prognostic implications of pretreatment albumin to C-reactive protein ratio in patients with hepatocellular carcinoma

**DOI:** 10.1186/s12885-019-5747-5

**Published:** 2019-06-04

**Authors:** Mian-Tao Wu, Su-Yin He, Shu-Lin Chen, Lin-Fang Li, Zheng-Qiang He, Yuan-Ying Zhu, Xia He, Hao Chen

**Affiliations:** 1 Sun Yat-sen University Cancer Center, State Key Laboratory of Oncology in South China, Collaborative Innovation Center for Cancer Medicine, Guangzhou, China; 2 Department of Laboratory Medicine, Sun Yat-sen University Cancer Center, Guangzhou, China; 3 State Key Laboratory of Ophthalmology, Zhongshan Ophthalmic Center, Sun Yat-sen University, Guangzhou, China

**Keywords:** Albumin to C-reactive protein ratio, Hepatocellular carcinoma, Overall survival, Disease-free survival

## Abstract

**Background:**

Despite recent advances in the treatments of hepatocellular carcinoma (HCC), the prognosis of HCC patients remains controversial. The purpose of this study was to investigate the prognostic performance of pretreatment albumin to C-reactive protein ratio (ACR) in patients with HCC.

**Methods:**

This study included 409 initially diagnosed HCC patients retrospectively. The optimal cut-off points for distinguishing high and low ACR value was determined by the X-tile software. The chi-squared test was used for comparing the baseline clinicopathologic parameters in different groups and subgroups. The Cox regression with log-rank tests was used to analyze OS and DFS, and Kaplan-Meier curves was used to estimate the prognosis of HCC patients.

**Results:**

Patients with lower ACR were significantly correlated with advanced clinical parameters, using a cut-off points of 5.4 (high ACR, *n* = 236 vs. low ACR, *n* = 173). Multivariate analysis demonstrated that ACR was associated with OS (HR = 0.544, 95% CI: 0.385–0.769, *p* = 0.001), with DFS (HR = 0.550, 95% CI: 0.392–0.772, *p* = 0.001). Treatment exposure (HR = 2.191; 95% CI: 1.533–3.132; *p* <  0.001), tumor size (HR = 1.973; 95% CI: 1.230–3.164; *p* = 0.005), serum AFP level (HR = 1.752; 95% CI: 1.277–2.403; *p* = 0.001), and TNM stage (HR = 0.470; 95% CI: 0.319–2.504; *p* <  0.001), were independent factors for OS in HCC patients. Treatment exposure (HR = 2.244; 95% CI: 1.590–3.166; *p* <  0.001), TNM stage (HR = 2.075; 95% CI: 1.436–3.000; *p* <  0.001), serum AFP level (HR = 1.819; 95% CI: 1.340–2.469; *p* = 0.001), tumor size (HR = 1.730; 95% CI: 1.113–2.689; *p* = 0.015), and ACR (HR = 0.550; 95% CI: 0.392–0.772; *p* = 0.001) were independent factors for DFS in HCC patients.

**Conclusions:**

Pretreatment ACR is a convenient and useful parameter for HCC patients predicting OS and DFS. Lower ACR was associated with advanced TNM stage, larger tumor size, and a high concentration of AFP. These results may help to design strategies to personalize management approaches among HCC patients.

## Background

Hepatocellular carcinoma (HCC) is the major histological subtype of primary liver cancer, and is one of the most common causes of cancer-related death worldwide, accounting for 788,000 deaths in 2015 [1]. Due to the initial symptoms of HCC are not obvious, many patients are in advanced stage when they are diagnosed, which leads to a poor prognosis. In the past decades, more and more attention has been paid to the relationship between inflammatory factors and tumors, many inflammation-based prognostic models had been reported in clinical oncology [2, 3]. Factors such as hepatitis B or C virus, aflatoxin, alcoholic or non-alcoholic cirrhosis, and smoking are all associated with the occurrence of HCC. In southeast Asian countries, cirrhosis caused by HBV infection is one of the main risk factors for HCC, from chronic hepatitis B to cirrhosis, and then to HCC. It has been found that immune inflammatory response is an important pathological process of tumor development, immune status and chronic inflammation play a significant role in promoting tumor recurrence and metastasis, which is closely related to poor prognosis. HCC is characterized by chronic systemic inflammation due to its unique etiological link with chronic hepatitis, which is related with the release of circulating inflammatory cytokines [4]. In recent years, a series of indicators are often reported in the prognosis of HCC, such as platelet / lymphocyte ratio (PLR), inflammation-based index (IBI), neutrophil / lymphocyte ratio (NLR), and the systemic immune-inflammation index [5–8].

C-reactive protein (CRP) is an acute phase protein, secreted by hepatocytes, can reflect the inflammation of the body under the regulation of pro-inflammatory factors [9]. Previous studies have demonstrated that high levels of CRP are associated with poor prognosis in many malignancies, including lung cancer, breast cancer, castration-resistant prostate cancer, gastric cancer, and colorectal cancer [10–14]. Albumin, also synthesized by hepatocytes, can reflect not only the nutritional status, but also the liver function. Albumin, is another important independent prognostic factor for many cancers, including HCC [15]. The decrease in albumin concentration is often caused by progressive weight loss and cachexia. Studies have shown that albumin levels in HCC patients are significantly lower than those in non-HCC patients, which may be related to liver dysfunction, HCC cell growth or liver damage caused by systemic inflammation [16]. It is generally considered that the treatment options are affected by tumor stage and liver function. Serum albumin, as one of the five elements of the Child-Pugh classification of liver function, is associated with the BCLC staging system [17]. The purpose of this study was to investigate the role of albumin to C-reactive protein ratio (ACR) as a significant and independent factor for HCC prognosis, and its relationship with clinical outcomes.

## Methods

### Patients

**Fig. 1:**
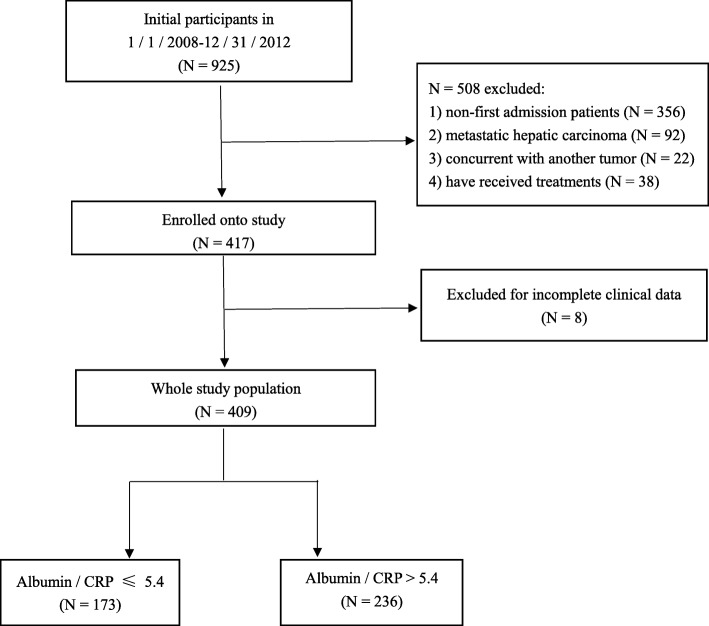
Flow chart of study participants

The current study has enrolled 925 participants from January 2008 to December 2012. There were 356 patients excluded due to non-first admission, 92 patients were metastatic hepatic carcinoma, 22 patients were concurrent with another tumor, 38 patients have received treatments in other hospitals. Eight additional patients were excluded due to incomplete clinical data. Thus, 409 newly diagnosed and untreated HCC patients were finally included. (Fig. 1.) Patient clinical data, including age, gender, diagnosis, smoke exposure, alcohol exposure, family history of cancer, treatment exposure, tumor size, TNM stage, date of the surgery, date of death, hepatitis B surface antigen (HBsAg), alpha-fetoprotein (AFP), C-reactive protein (CRP), albumin (ALB), alanine aminotransferase (ALT), aspartate aminotransferase (AST), blood platelet (PLT), absolute neutrophil count (NEUT), absolute lymphocyte count (LYMPH), neutrophil-to-lymphocyte ratio (NLR), platelet -to-CRP ratio (PCR), albumin-to-CRP ratio (ACR), and follow-up results were collected and calculated. Clinical staging of HCC was determined by TNM classification system [18]. The inclusion criteria for clinical cases were as follows: 1) have a clear histological or cytological diagnosis of hepatocellular carcinoma, 2) adequate clinicopathological and follow-up data, 3) routine blood examination were performed after admission and without preoperative adjuvant therapy, and 4) staged on the basis of TNM staging system. The exclusion criteria were as follows: 1) concurrent with other tumors or other types of liver cancer, 2) had prior treatments, 3) patients whose pretreatment laboratory data were not available, or without complete clinical data such as the TNM stage.

### Ethics statement

The design of this study was approved by the Sun Yat-sen University Cancer Center research ethics committee. All clinical data involved in this study were informed by the patient’s consent. The authenticity of this study has been validated by uploading the key raw data onto the Research Data Deposit public platform (www.researchdata.org.cn), with the approval RDD number as RDDA2017000272.

### Patient follow-up

All patients were followed up by telephone, out-patient and medical records after initial treatment. All cases were followed until June 30, 2016. The prognostic endpoints of this study were OS (overall survival) and DFS (disease-free survival). The endpoint of OS was defined as the death from HCC. The end-point of DFS was defined as recurrence or death from any causes. The total survival time was from the diagnosis to the end of the follow-up or at the end of the event. Patients still survive at the time of the last follow-up, or follow-up shedding of any cases for any reasons were defined as censored.

### Statistical analysis

All data were analyzed by SPSS19.0 software (IBM, Chicago, IL, USA). The X-tile software (version: 3.6.1, Copyright Yale University 2003–2005) was applied to determine the optimal cutoff points for the CRP, NLR, PCR, ACR. The baseline clinicopathologic features in different subgroups were compared by Pearson’s Chi-square test (Fisher’s exact test). Kaplan-Meier analysis was used to compare the overall survival rate and disease-free survival rate between groups or subgroups, and the difference was compared by log-rank test. Cox proportional hazard regression model with univariate and multivariate analysis was used to explore the prognostic factors. A *p* <  0.05 was considered significant statistically in this study.

## Results

### Definition of cut-off value

**Fig. 2:**
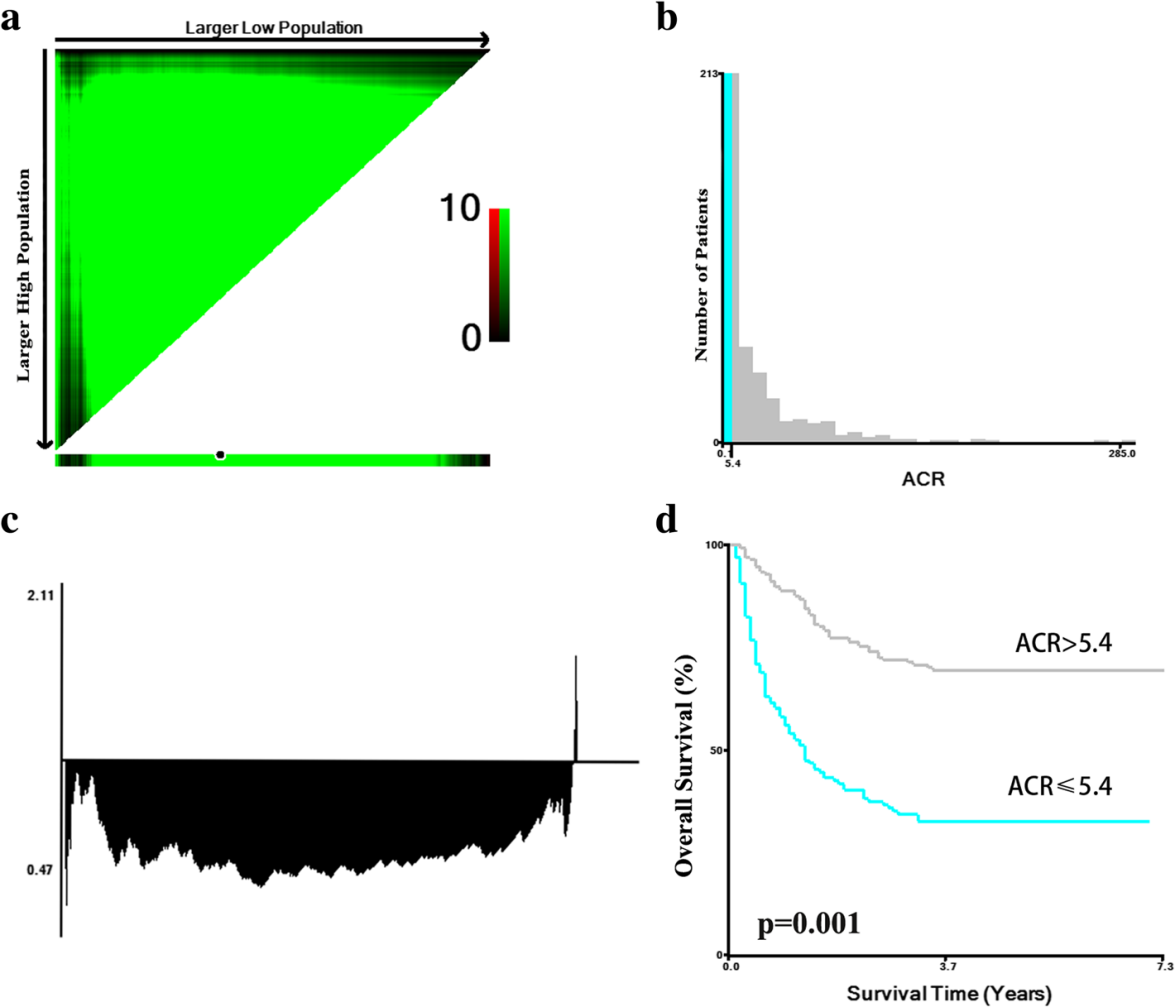
X-tile plots of the ACR on HCC patients. The plot shows the χ2 log-rank values that were created when the cohort was divided into two populations. The cutoff point, which is highlighted by a black/ white circle (**a**), is demonstrated on a histogram of the entire cohort (**b**), the relative risks for all cutoff points from low to high (left to right, x-axis), are calculated as event in high population / event risk in low population (**c**), a Kaplan-Meier overall survival curve (**d**). The preoperative ACR was divided at the optimal cutoff point, as defined by the most significant point on the plot (> 5.4 and ≤ 5.4, *p* = 0.001)

We applied the X-tile software [19] to determine the optimal cut-off value of Albumin / CRP ratio for OS and DFS, and the patients were classified into low ACR (< 5.4, *n* = 173) and high ACR groups (≥ 5.4, *n* = 236) (Fig. 2). The results demonstrate that a value of 5.4 had the most significant predictive value for OS (*p* <  0.0001, log-rank chi-square value = 65.1941, the relative risk of low ACR / high ACR: 2.08 / 1.00). Meanwhile, the optimal cut-off values of CRP, NLR, and PCR were also defined by the X-tile software. The optimal cut-off values of AFP, ALB, ALT, AST, and PLT were defined with medical decision level or medical reference range.

### Patient characteristics

The clinical characteristics and demographics of the 409 HCC cases were shown in Table 1. The majority of the patients included in this study were male (*n* = 369, 90.2%). In the low ACR group, there were 157 patients were males (38.4%) and 16 were females (3.9%), the median age was 50 (range 24–87) years. Seventy-seven cases (18.8%) with smoking history, 56 cases (13.7%) had a history of alcohol exposure, and 37 cases (9.0%) have a family history of cancer. One hundred and fifty-one patients (36.9%) with hepatitis B surface antigen positive. While in the high ACR group, 212 patients (51.8%) were males and 24 (5.9%) were females, the median age was 53 (range 13–94) years. Ninety-five cases (23.2%) had a history of smoking, 69 cases (16.9%) had a history of alcohol exposure, and 58 cases (14.2%) had a family history of cancer. Two hundred and five patients (50.1%) with hepatitis B surface antigen positive.

**Table 1:** Patient Demographics and Clinical Characteristics (*n* = 409) (Chi-square test; Fisher’s exact test)

Characteristics	ACR ≤ 5.4		ACR > 5.4		χ^2^	*P* value
	No.	%	No.	%		
Age (years)						
≤ 50	89	21.8	100	24.4	3.305	0.072
> 50	84	20.5	136	33.3		
Gender						
male	157	38.4	212	51.8	0.096	0.867
female	16	3.9	24	5.9		
Smoke exposure						
yes	77	18.8	95	23.2	0.741	0.418
no	96	23.5	141	34.5		
Alcohol exposure						
yes	56	13.7	69	16.9	0.462	0.516
no	117	28.6	167	40.8		
Family history of cancer						
yes	37	9.0	58	14.2	0.569	0.479
no	136	33.3	178	43.5		
Treatment exposure						
Hepatic resection	59	14.4	134	32.8	20.596	< 0.001
Other	114	27.9	102	24.9		
HBsAg						
positive	151	36.9	205	50.1	0.016	1.000
negative	22	5.4	31	7.6		
Tumor size						
< 5 cm	21	5.1	131	32.0	80.404	< 0.001
≥ 5 cm	152	37.2	105	25.7		
TNM						
I II	61	14.9	170	41.6	54.919	< 0.001
III IV	112	27.4	66	16.1		
AFP						
< 400 ng / mL	93	22.7	155	37.9	5.943	0.018
≥ 400 ng / mL	80	19.6	81	19.8		
CRP						
< 6.7 mg / L	1	0.2	228	55.7	373.600	< 0.001
≥ 6.7 mg / L	172	42.1	8	2.0		
ALB						
< 40 g / L	113	27.6	67	16.4	55.244	< 0.001
≥ 40 g / L	60	14.7	169	41.3		
ALT						
< 40 U / L	62	15.2	108	26.4	4.048	0.054
≥ 40 U / L	111	27.1	128	31.3		
AST						
< 45 U / L	80	19.6	114	27.9	0.170	0.690
≥ 45 U / L	93	22.7	122	29.8		
PLT						
< 100 *10ˆ9 / L	19	4.6	40	9.8	2.879	0.117
≥ 100 *10ˆ9 / L	154	37.7	196	47.9		
NLR						
< 2.3	85	20.8	125	30.6	0.587	0.484
≥ 2.3	88	21.5	111	27.1		
PCR						
< 47.8	172	42.1	60	14.7	222.671	< 0.001
≥ 47.8	1	0.2	176	43.0		

List of Abbreviations: *HBsAg* hepatitis B surface antigen, *AFP* alpha-fetoprotein, *CRP* C-reactive protein, *ALB* albumin, *ALT* alanine aminotransferase, *AST* aspartate aminotransferase, *PLT* platelet, *NLR* neutrophil-to-lymphocyte ratio, *PCR* platelet -to-CRP ratio, *ACR* albumin-to-CRP ratio

When it comes to treatment options, 193 patients (47.2%) underwent hepatic resection, while the others (*n* = 216, 52.8%) were given other treatment options, such as transcatheter arterial chemoembolization (TACE, *n* = 173, 42.3%), radiofrequency ablation (RFA, *n* = 23, 5.6%) and chemotherapy (*n* = 20, 4.9%). Two hundred and fifty-seven patients (62.8%) had larger tumor size (≥ 5 cm), 178 patients (43.5%) with an advanced stage of HCC. Significant differences can be seen between the two subgroups in treatment exposure (hepatic resection vs. others), in tumor size (< 5 cm vs. ≥ 5 cm), in TNM stage (I, II vs. III, IV), in serum AFP level (< 400 ng / mL vs. ≥ 400 ng / mL), in serum CRP level (< 6.7 mg / L vs. ≥ 6.7 mg / L), in serum albumin level (< 40 g / L vs. ≥ 40 g / L), and in platelet / CRP (< 47.8 vs. ≥ 47.8).

### Survival analysis

**Fig. 3:**
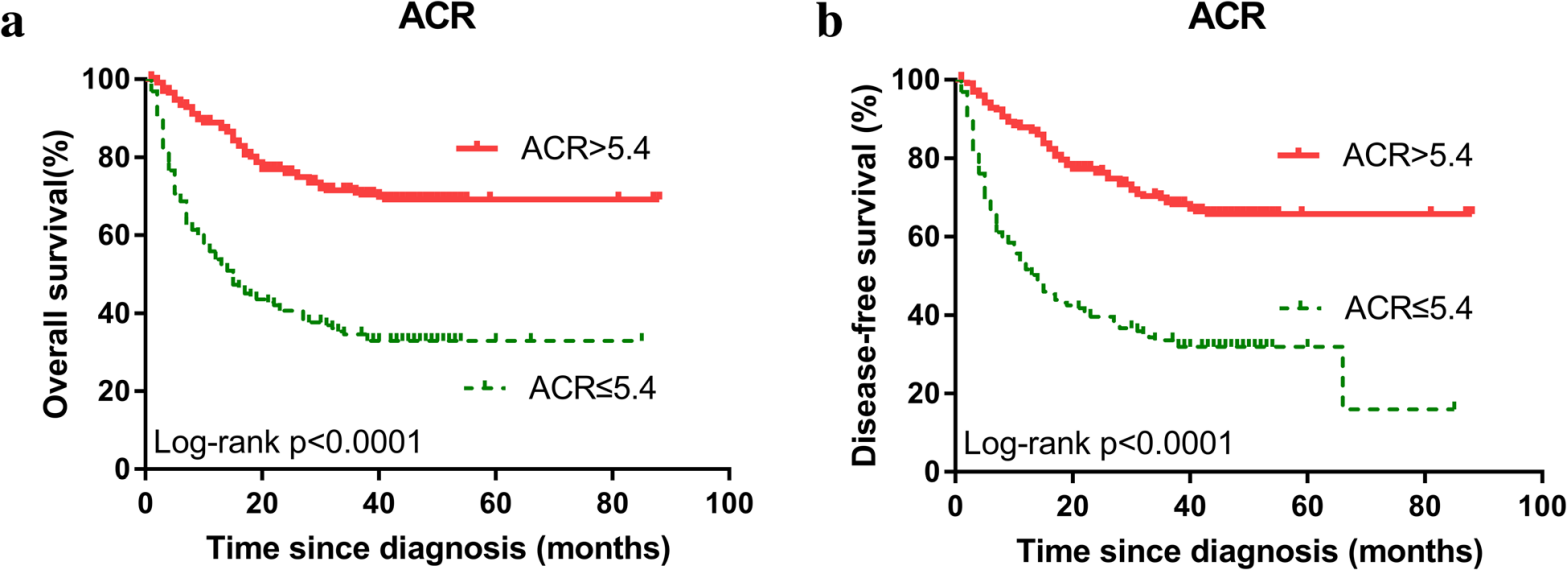
Kaplan-Meier estimated the OS (**a**) and DFS (**b**) for all HCC patients according to the pretreatment ACR. Abbreviation: ACR, albumin / CRP ratio

Among the 409 HCC patients, there were 166 cases died during follow-up, including 165 patients died of HCC, 1 patient died of massive hemorrhage of the digestive tract. One patient had received liver transplantation during follow-up; however, it turns out to die of HCC. The mean OS of the low ACR group was 35.060 months (95% CI, 29.217–40.904 months) vs. the high ACR group, 65.930 months (95% CI, 61.447–70.414 months). The 1-, 3-, and 5-year OS rate were 53.6, 34.5, and 32.7% for the low ACR group, respectively, and were 88.9, 70.9 and 69.1% for the high ACR, respectively. The Kaplan–Meier analysis assessed the OS and DFS for all patients of two groups were shown in Fig. 3. The results showed a significant correlation between high ACR group and longer OS (*p* <  0.0001), which indicated that patients with high ALB and low CRP level had a better prognosis.

### Univariate and multivariate analyses for OS and DFS

Univariate and multivariate Cox proportional hazards regression of prognostic factors for OS were shown in Table 2. In the univariate analysis, the significant factors were tumor size (HR = 4.332; 95% CI: 2.896–6.482; *p* <  0.001), TNM stage (HR = 0.211; 95% CI: 0.152–0.293; *p* <  0.001), treatment exposure (HR = 3.005; 95% CI: 2.157–4.188; *p* <  0.001), AFP (HR = 2.297; 95% CI: 1.691–3.122; *p* <  0.001), CRP (HR = 3.378; 95% CI: 2.460–4.640; *p* <  0.001), ALB (HR = 0.665; 95% CI: 0.490–0.902; *p* = 0.009), PCR (HR = 0.323; 95% CI: 0.229–0.457; *p* <  0.001), and ACR (HR =0.302; 95% CI: 0.221–0.413; *p* <  0.001). The multivariate analysis (Likelihood ratio forward method), using the above clinical parameters, indicated that tumor size (HR = 1.973; 95% CI: 1.230–3.164; *p* = 0.005), TNM stage (HR = 0.470; 95% CI: 0.319–2.504; *p* <  0.001), treatment exposure (HR = 2.191; 95% CI: 1.533–3.132; *p* <  0.001), serum AFP level (HR = 1.752; 95% CI: 1.277–2.403; *p* = 0.001), and ACR (HR = 0.544; 95% CI: 0.385–0.769; *p* = 0.001) were independent and significant prognostic factors in HCC patients. Table 3 showed univariate and multivariate Cox proportional hazards regression of prognostic factors for DFS. In the univariate analysis, the significant factors were tumor size (HR = 3.739; 95% CI: 2.577–5.426; *p* <  0.001), TNM stage (HR = 4.396; 95% CI: 3.207–6.026; *p* <  0.001), treatment exposure (HR = 3.029; 95% CI: 2.195–4.181; *p* <  0.001), AFP (HR = 2.324; 95% CI: 1.727–3.128; *p* <  0.001), CRP (HR = 3.227; 95% CI: 2.376–4.381; *p* <  0.001), ALB (HR = 0.625; 95% CI: 0.465–0.841; *p* = 0.002), PCR (HR = 0.340; 95% CI: 0.244–0.473; *p* <  0.001), and ACR (HR = 0.315; 95% CI: 0.232–0.426; *p* <  0.001). According to the multivariate analysis using likelihood ratio forward method, treatment exposure (HR = 2.244; 95% CI: 1.590–3.166; *p* <  0.001), TNM stage (HR = 2.075; 95% CI: 1.436–3.000; *p* <  0.001), serum AFP level (HR = 1.819; 95% CI: 1.340–2.469; *p* = 0.001), tumor size (HR = 1.730; 95% CI: 1.113–2.689; *p* = 0.015), and ACR (HR = 0.550; 95% CI: 0.392–0.772; *p* = 0.001) were independent and significant prognostic factors for DFS in HCC patients.

**Table 2:** Univariate and multivariate Cox proportional hazards regression of prognostic factors for overall survival

Characteristics	Univariate			Multivariate		
	HR	95% CI	*P* value	HR	95% CI	*P* value
Age (years; ≤ 50 vs. > 50)	0.878	0.647–1.192	0.403			
Gender (male vs. female)	1.228	0.761–1.980	0.400			
Smoke exposure (yes vs. no)	0.972	0.713–1.324	0.857			
Alcohol exposure (yes vs. no)	1.055	0.759–1.465	0.751			
Family history of cancer (yes vs. no)	0.924	0.641–1.333	0.673			
Tumor size (< 5 cm vs. ≥ 5 cm)	4.332	2.896–6.482	< 0.001	1.973	1.230–3.164	0.005
TNM (I and II vs. III and IV)	0.211	0.152–0.293	< 0.001	0.470	0.319–0.693	< 0.001
Treatment exposure (Hepatic resection vs. others)	3.005	2.157–4.188	< 0.001	2.191	1.533–3.132	< 0.001
HBsAg (positive vs. negative)	0.820	0.536–1.256	0.362			
AFP (ng / mL; < 400 vs. ≥ 400)	2.297	1.691–3.122	< 0.001	1.752	1.277–2.403	0.001
CRP (mg / L; < 6.70 vs. ≥ 6.70)	3.378	2.460–4.640	< 0.001	0.907	0.329–2.504	0.851
ALB (g / L; < 40 vs. ≥ 40)	0.665	0.490–0.902	0.009	0.960	0.691–1.333	0.807
ALT (U / L; < 40 vs. ≥ 40)	1.178	0.862–1.610	0.304			
AST (U / L; < 45 vs. ≥ 45)	1.022	0.753–1.387	0.889			
PLT (*10ˆ9 / L; < 100 vs. ≥ 100)	1.314	0.823–2.097	0.253			
NLR (< 2.3 vs. ≥ 2.3)	0.963	0.709–1.307	0.808			
PCR (< 47.8 vs. ≥ 47.8)	0.323	0.229–0.457	< 0.001	0.886	0.506–1.551	0.672
ACR (≤ 5.4 vs. > 5.4)	0.302	0.221–0.413	< 0.001	0.544	0.385–0.769	0.001

List of Abbreviations: *HBsAg* hepatitis B surface antigen, *AFP* alpha-fetoprotein, *CRP* C-reactive protein, *ALB* albumin, *ALT* alanine aminotransferase, *AST* aspartate aminotransferase, *PLT* platelet, *NLR* neutrophil-to-lymphocyte ratio, *PCR* platelet -to-CRP ratio, *ACR* albumin-to-CRP ratio

**Table 3:** Univariate and multivariate Cox proportional hazards regression of prognostic factors for disease-free survival

Characteristics	Univariate			Multivariate		
	HR	95% CI	*P* value	HR	95% CI	*P* value
Age (years; ≤ 50 vs. > 50)	0.941	0.699–1.267	0.690			
Gender (male vs. female)	1.209	0.759–1.927	0.424			
Smoke exposure (yes vs. no)	1.021	0.757–1.376	0.894			
Alcohol exposure (yes vs. no)	1.184	0.865–1.620	0.292			
Family history of cancer (yes vs. no)	0.862	0.599–1.239	0.422			
Tumor size (< 5 cm vs. ≥ 5 cm)	3.739	2.577–5.426	< 0.001	1.730	1.113–2.689	0.015
TNM (I and II vs. III and IV)	4.396	3.207–6.026	< 0.001	2.075	1.436–3.000	< 0.001
Treatment exposure (Hepatic resection vs. others)	3.029	2.195–4.181	< 0.001	2.244	1.590–3.166	< 0.001
HBsAg (positive vs. negative)	0.819	0.540–1.242	0.346			
AFP (ng / mL; < 400 vs. ≥ 400)	2.324	1.727–3.128	< 0.001	1.819	1.340–2.469	< 0.001
CRP (mg / L; < 6.70 vs. ≥ 6.70)	3.227	2.376–4.381	< 0.001	0.863	0.318–2.342	0.772
ALB (g / L; < 40 vs. ≥ 40)	0.625	0.465–0.841	0.002	0.910	0.662–1.251	0.561
ALT (U / L; < 40 vs. ≥ 40)	1.169	0.864–1.583	0.312			
AST (U / L; < 45 vs. ≥ 45)	0.974	0.724–1.309	0.860			
PLT (*10ˆ9 / L; < 100 vs. ≥ 100)	1.325	0.840–2.091	0.226			
NLR (< 2.3 vs. ≥ 2.3)	0.932	0.693–1.253	0.640			
PCR (< 47.8 vs. ≥ 47.8)	0.340	0.244–0.473	< 0.001	0.821	0.479–1.409	0.474
ACR (≤ 5.4 vs. > 5.4)	0.315	0.232–0.426	< 0.001	0.550	0.392–0.772	0.001

List of Abbreviations: *HBsAg* hepatitis B surface antigen, *AFP* alpha-fetoprotein, *CRP* C-reactive protein, *ALB* albumin, *ALT* alanine aminotransferase, *AST* aspartate aminotransferase, *PLT* platelet, *NLR* neutrophil-to-lymphocyte ratio, *PCR* platelet -to-CRP ratio, *ACR* albumin-to-CRP ratio

### Subgroup analysis for OS and DFS

**Fig. 4:**
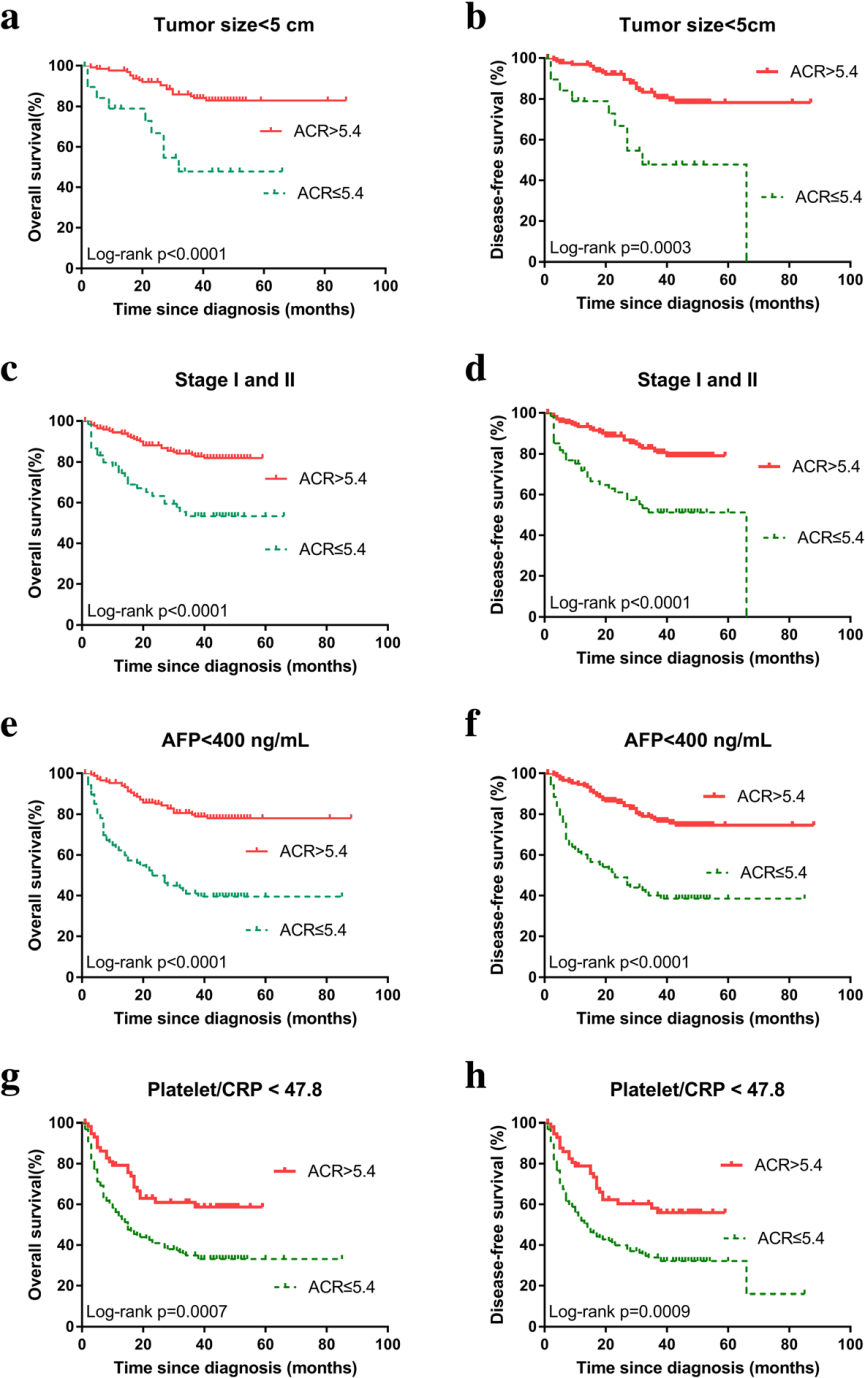
Survival outcomes in patients with high (> 5.4) and low (≤ 5.4) ACR. **a** OS rate in the subgroup of tumor size < 5 cm. **b** DFS rate in the subgroup of tumor size < 5 cm. **c** OS rate in the subgroup of early stage (I or II). **d** DFS rate in the subgroup of early stage (I or II). **e** OS rate in the subgroup of AFP <  400 ng / mL. **f** DFS rate in the subgroup of AFP <  400 ng / mL. **g** OS rate in the subgroup of platelet / CRP <  47.8. **h** DFS rate in the subgroup of platelet / CRP <  47.8

**Fig. 5:**
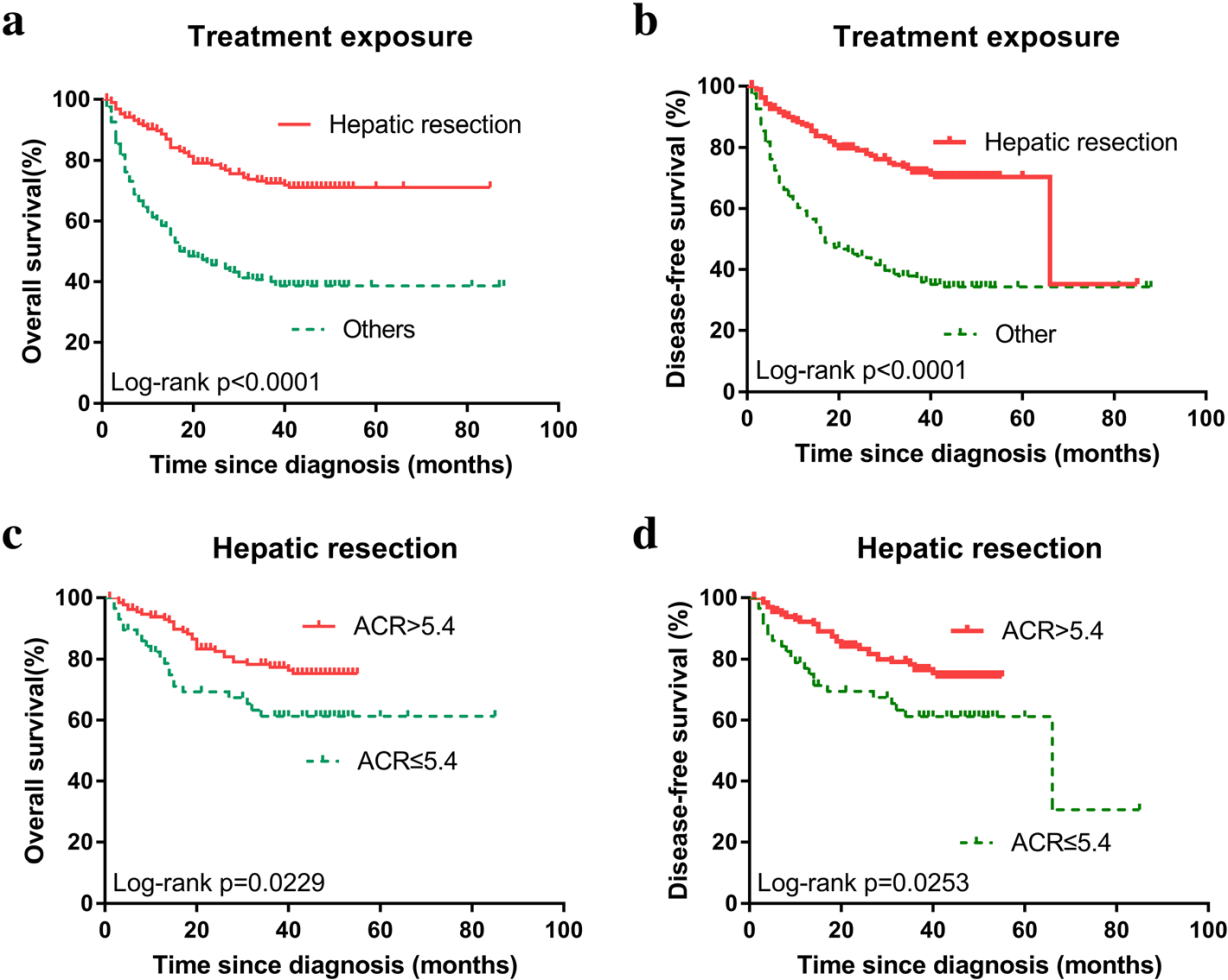
Kaplan–Meier estimates of overall survival according to treatment exposure. Patients underwent hepatic resection had a significantly longer OS (**a**.), DFS (**b**) than those accepted other treatments (*p* <  0.0001). **c** OS rate in the subgroup of hepatic resection. **d** DFS rate in the subgroup of hepatic resection

Notably, as albumin / CRP ratio was significant associated with other parameters, we therefore performed subgroup analysis according to tumor size, TNM stage, treatment exposure, serum AFP level, and platelet / CRP (platelet to CRP ratio) (Fig. 4). The 1-, 3-, and 5-year OS rate of the high ACR group were significantly higher than those of the low ACR group in tumor size < 5 cm subgroup (97.6, 85.0, 82.9% vs. 78.9, 47.8, 47.8%, respectively; *p* <  0.0001) (Fig. 4a), in early stage (stage Iand II) subgroup (94.5, 83.5, 81.9% vs. 74.4, 53.2, 53.2%, respectively; *p* <  0.0001) (Fig. 4c), in serum AFP level <  400 ng/mL subgroup (95.4, 79.8, 77.9% vs. 62.2, 40.8, 39.4%, respectively; *p* <  0.0001) (Fig. 4e), and in platelet / CRP <  47.8 subgroup (79.2, 61.0, 58.7% vs. 54.1, 34.9, 33.2%, respectively; *p* = 0.0007) (Fig. 4g). The 1-, 3-, and 5-year DFS rate of the high ACR group were significantly higher than those of the low ACR group in tumor size < 5 cm subgroup (96.9, 81.6, 78.3% vs. 78.9, 47.8, 47.8%, respectively; *p* = 0.0003) (Fig. 4b), in early stage (stage Iand II) subgroup (93.3, 81.4, 79.1% vs. 71.8, 51.2, 51.2%, respectively, *p* <  0.0001) (Fig. 4d), in serum AFP level <  400 ng / mL subgroup (94.7, 77.5, 74.7% vs. 60.2, 40.0, 38.6%, respectively, *p* <  0.0001) (Fig. 4f), and in platelet / CRP <  47.8 subgroup (78.9, 58.2, 55.9% vs. 51.9, 33.8, 32.1%, respectively, *p* = 0.0009) (Fig. 4h). However, Kaplan–Meier analyses and log-rank tests demonstrated the OS rate and DFS rate of all HCC patients underwent hepatic resection significantly higher than those accepted other treatment options (both *p* <  0.0001) (Fig. 5a, b). In hepatic resection subgroup, the 1-, 3-, and 5-year OS rate of the high ACR group were significantly higher than those of the low ACR group (93.8, 77.3, 75.2% vs. 80.5, 61.2, 61.2%, respectively, *p* = 0.0229) (Fig. 5c), the 1-, 3-, and 5-year DFS rate of the high ACR group were significantly higher than those of the low ACR group (92.3, 76.5, 74.4% vs. 77.0, 61.2, 61.2%, respectively, *p* = 0.0253) (Fig. 5d).

## Discussion

Prognosis assessment is an important step in HCC management. Although treatment options have been made significant progress, the prognosis of HCC patients still remains substandard. Any attempts to evaluate prognosis should take the tumor stage, the degree of liver function damage, and the presence of tumor-related symptoms into account [20]. Systemic inflammatory response was considered to be essential factor in the development of malignancies. As one of the acute phase proteins produced by the hepatocytes, CRP has many biological functions, Jun et al. have expounded that a high serum CRP level was associated with larger tumor lesions in patients with HCC [21]. In addition, Kim et al. had indicated that serum CRP levels on the fourth day was the best predictor of postoperative complications after gastrectomy [13]. Albumin is the most important protein in plasma, which represents nutritional status and maintains osmotic pressure. Forrest et al. had revealed that high levels of CRP and hypoproteinemia could be used to evaluate the prognosis of 161 inoperable non-small cell lung cancer patients [22]. Ishizuka et al. had designed their study to assess the prognostic value of CRP / albumin ratio in patients with colorectal cancer, which could predict the survival rate after the colorectal resection [14]. With regard to hepatocellular carcinoma, Kinoshita et al. had retrospectively evaluated 186 HCC patients and revealed that the pretreatment CRP / albumin ratio might be a reliable prognostic factor [23]. However, the albumin / CRP ratio, also considered as the Inflammatory-Nutritional Index (INI), is seldom reported with a prognosis of cancer. The current study firstly investigated the clinical and prognostic implication of albumin / CRP ratio (ACR) in patients with HCC.

In this study, we retrospectively evaluated the albumin / CRP ratio as a prognostic factor in 409 newly diagnosed HCC patients. The results of Cox regression analysis suggest that the pretreatment ACR was an independent factor for HCC prognosis, high ACR is associated with better outcome in HCC patients. In the univariate analysis of OS and DFS, the significant prognostic parameters are tumor size, TNM stage, treatment exposure, serum AFP level, serum CRP level, serum albumin level, albumin / CRP ratio, and platelet / CRP ratio. In the multivariate analysis, the results indicated that tumor size, TNM stage, treatment exposure, serum AFP level, and albumin / CRP ratio were independent factors for HCC prognosis. The results indicated that treatment exposure, TNM stage, and tumor size, have higher HR value than ACR and AFP, which mean that they have a greater weight in representing its prognosis. However, these parameters were all subjective clinical features, while ACR and AFP were reliable markers that can be easily detected in peripheral blood. Accordingly, clinicians can make a preliminary judgment on the prognosis of HCC patients. We also analyzed the correlation between ACR and certain clinical parameters in subgroups. The results suggested that tumor size, TNM stage, treatment exposure, and serum AFP level are significantly correlated with ACR. Notably, patients underwent hepatectomy seem had a better prognosis of OS and DFS, compared with other treatment options, including TACE, RFA, and chemotherapy. This may explain that hepatectomy was the main treatment option for HCC patients without cirrhosis [20]. El-Serag HB et al. had revealed that hepatectomy will be a priority for patients with early-stage, presenting a diameter of less than 5 cm, or no more than three nodules, which is associated with an OS rate of 90% [24]. This also reveals that tumor size and TNM stage are correlated with OS.

Although previous studies have demonstrated that Neutrophil-to-Lymphocyte Ratio (NLR) was a reliable prognostic factor for OS in HCC patients [5, 25–27]. However, in the present study, no significant differences of NLR for OS in HCC patients was observed. As to ALT and AST, are commonly measured clinically as biomarkers for liver health. Our previous study has indicated that high preoperative AST concentrations can be used as prognostic factors for NSCLC when the cutoff value was 19 U / L [28]. Yet no association with the prognosis of HCC patients was shown in the present study. The possible explanations for the inconsistencies mentioned above may as follows: firstly, all the subjects involved in our study are Chinese, whereas other reports were different ethnic population; secondly, we performed the X-tile software to defined the optimal cut-off value, while other studies used ROC curves; last but not the least, the methods for detecting blood samples may be different, which could affect the results.

Serum AFP is currently the most commonly used as diagnosis and surveillance for hepatocellular carcinoma. However, serum AFP levels have low sensitivity (25 to 65%), when using a cutoff point of 20 ng / mL, for the detection of HCC. When it comes to HCC diagnosis, an AFP level of 400 ng / mL or higher also has great predictive value [29]. Despite its low sensitivity in the diagnosis of HCC, serum levels of AFP still served as an independent factor for HCC prognosis in our study. In the subgroup of AFP <  400 ng / mL, the 1-, 3-, and 5-year OS rate of the high ACR group were significantly higher than those of the low ACR group (95.4, 79.8, 77.9% vs 62.2, 40.8, 39.4%, respectively; *p* <  0.0001). However, An et al. demonstrated that pretreatment serum AFP with a cutoff point of 20 ng / mL served as a reliable predictor of prognosis among HCC patients underwent hepatectomy in a single-center cohort from China, which enrolled 251 HCC patients [30]. Therefore, whether serum AFP, with a cutoff point of 20 ng / mL, can serve as another independent factor of OS for HCC patients in our cohort need further investigation.

A major strength of this study is that it firstly analyzed the ACR as a significant and independent factor for OS and DFS, involving 409 newly diagnosed HCC patients, retrospectively. However, it still has some limitations. First, the majority of cases included in this study were HCC patients with hepatitis B virus infection, and consequently, these results may not be applicable to patient cohorts from Western countries, who are usually dominated by hepatitis C virus infection. Second, since it is a single-center cohort, this study needs a multi-center prospective design in a larger population to validate our results. Third, our study only analyzed the impact of some parameters on the OS and DFS in HCC patients, while other parameters such as prothrombin induced by vitamin K absence-II (PIVKA-II) [31], carcinoembryonic antigen (CEA) [32], the Barcelona Clinic Liver Cancer (BCLC) staging system [33], were not taken into account in this study.

## Conclusions

From the above discussion, the conclusion can be reached that pretreatment ACR was a significant and independent factor for OS and DFS in patients with HCC. Pretreatment ACR was correlated with tumor size, TNM stage, treatment exposure, and serum AFP levels. Due to the mechanism of ACR in HCC patients is not clear, further prospective analyses should be conducted with larger population or multi-center cohorts.

## Data Availability

The datasets used and analyzed during the current study are available from the corresponding author on reasonable request.

## Abbreviations

ACR: Albumin to C-reactive Protein Ratio
AFP: Alpha-fetoprotein
ALB: Albumin
ALT: Alanine aminotransferase
AST: Aspartate aminotransferase
BCLC: Barcelona Clinic Liver Cancer
CEA: Carcinoembryonic antigen
CRP: C-reactive protein
DFS: Disease-free survival
HBsAg: Hepatitis B surface antigen
HCC: Hepatocellular carcinoma
NLR: Neutrophil-to-lymphocyte ratio
PCR: Platelet to C-reactive protein ratio
PIVKA-II: Prothrombin induced by vitamin K absence-II
PLT: Platelet
RFA: Radiofrequency ablation
TACE: Transcatheter arterial chemoembolization
